# Heparin in malignant glioma: review of preclinical studies and clinical results

**DOI:** 10.1007/s11060-015-1826-x

**Published:** 2015-06-30

**Authors:** Rosalie Schnoor, Sybren L. N. Maas, Marike L. D. Broekman

**Affiliations:** Department of Neurosurgery, University Medical Center Utrecht, Heidelberglaan 100, 3584 CX Utrecht, The Netherlands; Brain Center Rudolf Magnus, University Medical Center Utrecht, Heidelberglaan 100, 3584 CX Utrecht, The Netherlands

**Keywords:** Glioblastoma multiforme, Glioma, Heparin, Low molecular weight heparin

## Abstract

Glioblastoma multiforme (GBM) is the most common primary brain tumor that is invariably lethal. Novel treatments are desperately needed. In various cancers, heparin and its low molecular weight derivatives (LMWHs), commonly used for the prevention and treatment of thrombosis, have shown therapeutic potential. Here we systematically review preclinical and clinical studies of heparin and LMWHs as anti-tumor agents in GBM. Even though the number of studies is limited, there is suggestive evidence that heparin may have various effects on GBM. These effects include the inhibition of tumor growth and angiogenesis in vitro and in vivo, and the blocking of uptake of extracellular vesicles. However, heparin can also block the uptake of (potential) anti-tumor agents. Clinical studies suggest a non-significant trend of prolonged survival of LMWH treated GBM patients, with some evidence of increased major bleedings. Heparin mimetics lacking anticoagulant effect are therefore a potential alternative to heparin/LMWH and are discussed as well.

**Electronic supplementary material:**

The online version of this article (doi:10.1007/s11060-015-1826-x) contains supplementary material, which is available to authorized users.

## Introduction

Glioblastoma multiforme (GBM) is the most common primary brain tumor and is without exception lethal. Despite (advances in) neurosurgery, radiation and chemotherapy, median survival still does not extend beyond 15 months, emphasizing a dire need for novel treatments [1]. Most GBM patients are treated peri-operatively with low molecular weight heparin (LMWH) for the prevention of thrombotic complications. While LMWH is a well-established drug for the prevention and treatment of thrombosis, it has regained interest as a potential anti-cancer agent. This interest in heparins as anti-cancer agents was ignited by the sub-analysis [2] (n = 129) of two trials published in 1992 [3, 4], indicating lower mortality rates among cancer patients receiving LMWH as opposed to heparin, a finding that was later disputed in a larger analysis (n = 672) that included brain tumor patients [5]. Interestingly though, a 2013 Cochrane meta-analysis found a significant survival benefit for LWMH/heparin treated patients after 24 months, but not after 12 months [6].

In vitro cancer studies indicate both heparin and LMWH to inhibit angiogenesis, invasion, and metastasis of solid tumors [7]. Moreover, the uptake of extracellular vesicles (EVs), 50–1000 nm membrane vesicles, implicated in GBM biology [8, 9], is blocked by heparin [10]. In animal models for different, non-GBM tumors, heparin [11] and LMWH [12] were shown to inhibit tumor growth and to prolong survival. The effect of heparin-based therapies on GBM tumors and thus its potential for GBM patients is currently unknown. Here we systematically review literature on the effects of heparin/LMWH on GBM in preclinical and clinical settings (Supplementary Table 1 for search terms and Supplementary Fig. 1 for flow-chart).

## Pharmacodynamic properties of heparin

Unfractionated heparin (UFH, here; heparin) is a highly sulfated glycosaminoglycan (GAG), closely related to heparan sulfate, which binds to a range of target molecules and can subsequently affect their activity [13]. Heparin is produced endogenously by basophils and mast cells, and can be found in a variety of organs. The GAG chains in heparin each contain 200–300 saccharide units, resulting in a variable molecular weight. Heparin, isolated from mucosa of animals, is the most widely used anticoagulant in the world [14]. It used to be the drug of choice for the prevention and treatment of venous thromboembolism (VTE), but since 1999 it has been replaced by LMWHs [15]. LMWH variants such as dalteparin, nadroparin and tinzaparin are heparin fragments with less than 18 saccharide units per GAG chain and a molecular mass of approximately 5000 Da. The anticoagulant activity of heparin and LMWHs is indirect and largely based on binding of heparin or heparin fragments to antithrombin 3 [16], a plasma protease inhibitor with the ability to inactivate several enzymes of the coagulation cascade, including factors X and II. Heparin also promotes tissue factor pathway inhibitor (TFPI) by neutralizing the effects of tissue factor (TF), a high affinity receptor for coagulation factor VII and therefore an important initiator of the coagulation cascade [17]. Interestingly, TF has been implicated in glioma biology and its expression seems to be related to molecular subtype and to mutations in EGFR and other genes implicated in GBM [18]. Moreover, TF was shown to be the driver of growth activation of dormant GBM cells in an in vivo model [19] and increased expression of TF is found on microparticles in GBM patients [20].

Specific non-anticoagulant effects have been ascribed to heparin as well. It downregulates the inflammatory response by binding immune-activating enzymes and inhibits adhesion of leukocytes to the endothelial wall [21]. Several animal studies and case reports also suggest a beneficial effect on wound healing and tissue repair [22]. However, most research into non-anticoagulant effects of heparin has focused on its impact in cancer [7, 23, 24]. GBM specific studies will be discussed below.

## Preclinical data

### Angiogenesis

One of the hallmarks of GBM is angiogenesis and numerous factors have been shown to play an important role in this process [25, 26]. Heparin and LMWH influence angiogenesis by affecting some of these factors [27–31] as discussed below.

Tumor-derived adhesion factor (TAF), also known as mac25, is expressed in normal brain, lung, and muscle, but also in various human cancer tissues [27]. In GBMs, TAF is localized specifically in small blood vessels near tumor cells. In vitro it co-localizes with collagen IV and is found in tube-like structures of endothelial cells, indicating a role in angiogenesis. TAF is a heparin binding protein and heparin (10μg/ml) inhibits binding of TAF to endothelial cells [27]. High concentrations of heparin (20μg/ml) prevent the formation of tube-like structures by endothelial cells, indicating the ability of heparin to inhibit early steps of angiogenesis.

Secondly, heparin and tinzaparin (an LMWH) reduce endothelial cell proliferation in a dose-dependent manner in vitro [28]. Heparin was shown to bind to heparan sulfate proteoglycans (HSPGs), preventing the ability of HSPGs to act as co-receptors for pro-angiogenic factors and antagonizing proliferation in this way.

Hypoxia, an important feature of GBMs, has been shown to influence several biological processes, including neovascularization (mediated by vascular endothelial growth factor; VEGF) and activation of the coagulation system (mediated by expression of TF) [32, 33]. Protease-activated receptor 2 (PAR-2), a G-protein coupled receptor active in coagulation dependent signaling, is up-regulated by hypoxia and TF [34]. The induction of PARs was found to activate heparin binding EGF-like growth factor (HB-EGF), a pro-angiogenic growth factor [29]. Heparin can inhibit HB-EGF activity through interference with HSPG binding, and also reverse PAR-2 dependent proliferation of endothelial cells, thereby inhibiting GBM neovascularization [29].

Moreover, in a U87-MG GBM xenograft mouse model it has been shown that heparin can bind to hepatocyte growth factor/scatter factor (HGF/SF), which plays a role in tumorigenesis and angiogenesis and is expressed in GBM [35]. Blocking of HGF/SF with heparin and a neutralizing HGF antibody resulted in reduced tumor burden due to decreased angiogenesis in vivo [30].

SU5416 is a tyrosine kinase receptor inhibitor that inhibits both vascular endothelial growth factor receptor 2 (VEGFR-2) and c-kit [31]. Subsequently, this drug has been shown to reduce vascular density in GBM [36]. In vivo, combined treatment with SU5416 and dalteparin (an LMWH) enhanced the inhibition of tumor growth by SU5416, whereas dalteparin alone did not result in reduced tumor growth [31]. A possible explanation for this observation could be competitive binding of VEGF by LMWH and SU5416. Combining LMWH with a VEGFR-2 inhibitor (i.e. SU5416) could thereby promote the anti-angiogenic effect of SU5416.

### Extracellular vesicles

Recently, accumulating evidence suggests that EVs, 50–1000 nm membrane vesicles released by all cell types, play a role in tumor biology [8, 37]. For instance, EVs derived from GBM cells have been shown to have a stimulating effect on neovascularization, tumor cell growth [8], and to modify monocytic cells [38]. EVs can be taken up by recipient cells and transfer tumor-derived contents, including functional RNAs, miRNAs and protein [9, 39]. Blocking the uptake of EVs has gained interest as a possible anti-cancer strategy. Recently, heparin has been shown to block transfer of EVs into cells [10]. A heparin concentration of 0.1 μg/ml resulted in a 90 % reduction in EV uptake into U87-MG glioma cells in an in vitro co-culture system. Other work showed that HSPGs on the recipient cell surface act as a receptor for EVs, and that these HSPGs can be inhibited in a dose dependent manner by heparin or other HS mimetics [40]. These data suggest that heparin interacts with tumor-derived EVs and blocks attachment of EVs to recipient cells, which could possibly result in an anti-tumor effect.

### Extracellular matrix

Connecting to and modifying extracellular matrix (ECM) proteins is crucial for survival and migration of (glioma) cells [41]. Heparin has been shown to inhibit GBM cell-attachment to laminin and fibronectin, two ECM proteins [42]. A different study did not demonstrate enoxaparin (an LMWH) to have a significant inhibitory effect on migration of tumor cells in culture; tumor cell proliferation was however inhibited [43]. This is not in line with what others have observed, as Okumura and co-workers found an increase of tumor cell proliferation by exposure of cells to heparin [44]. Unfortunately, different cell culture conditions, such as the presence of ECM or basic fibroblast growth factor (bFGF), make it impossible to draw definite conclusions.

### Interaction with (potential) therapeutics

Targeted drug delivery for GBM treatment has gained interest [45]. However, the delivery method has to meet several requirements, such as bypassing the immune system, crossing the blood–brain barrier, and selectively targeting GBM cells. Low-density lipoprotein (LDL), an endogenous carrier of cholesterol, could potentially meet these requirements and was tested as a drug carrier targeting GBM cells. LDL is of particular interest for drug delivery, since LDL-receptor activity is increased in dividing cells. LDL carrying the drug aclacinomycin A (I-LDL-aclacinomycin A) was found to reduce tumor cell growth in vitro [46]. The presence of heparin however, inhibited receptor-mediated uptake of I-LDL-aclacinomycin A in a glioblastoma cell line [47], indicating that heparin might inhibit receptor-mediated uptake and degradation of LDL by tumor cells.

The same effect was observed when a viral vector was used to deliver genes encoding anti-tumor proteins to GBM cells. Using an AAV library to select capsid variants, a new chimeric AAV vector was created that was able to successfully transduce a multitude of glioma cell lines [48]. In fact some serotypes of AAV (2, 3, 6 and 13) enter cells via heparin binding [49]. Incubation of cells with the viral vector in the presence of heparin can therefore greatly reduce the transduction efficiency. Taking these results into account, the combination of heparin and targeted drug delivery such as LDL or an AAV vector could prove to be counteractive.

## Clinical data

Three studies describe the effect of heparin and/or LMWHs on survival in human GBM patients [50–52].

In 2002, the Eastern Cooperative Oncology Group (ECOG) initiated a controlled trial to investigate if LMWH treatment, in combination with radiation therapy, could improve overall survival (OS) in newly diagnosed GBM patients [50]. The patient population for this trial consisted of 42 supratentorial GBM patients with an estimated expected survival of at least 8 weeks and an ECOG performance status of 0–2. On the first day of radiotherapy, LMWH (dalteparin) was introduced daily for a planned 24 months or until progression of disease, at a dose of 5000 IE subcutaneously which is considered a prophylactic dose for VTE [15]. After first progression, patients could continue dalteparin therapy in addition to standard regimens. A comparable group of 72 patients was selected from the Radiation Therapy Oncology Group (RTOG) GBM database to serve as historical controls. Median survival was 11.9 months in the trial group, a non-significant improvement (P value of 0.47) compared to the RTOG database cohort. Within the study group, a subgroup of four patients who continued dalteparin after first (radiological) progression had a median survival of 7.9 months, compared to 3 months in the group who stopped LMWH treatment. The study closed early as the original recruitment goal seemed unrealistic after the introduction of temozolomide as standard of care for GBM patients in 2004 [50].

The PRODIGE trial, a randomized placebo-controlled trial, studied the effect of long term subcutaneous LMWH (dalteparin) treatment in patients with newly diagnosed high grade glioma (WHO grade 3 or 4) [51]. Primary endpoints were documented symptomatic deep venous thrombosis (DVT) or pulmonary embolism occurring during the 6 months post-randomization; secondary endpoints were OS and hemorrhage. The treatment group received 5000 IE dalteparin subcutaneously daily, control glioma patients were injected with saline. The trial faced difficulties recruiting patients and was terminated early because of insufficient study drug quantity and a trend towards increased incidence of major bleeding in patients who received LMWH. A total of 186 patients were randomized, treated and analyzed. Long-term treatment with LMWH did not result in improved survival rates, as the 12-month mortality rates were 47.8 % for LMWH+ and 45.4 % for placebo patients, a non-significant difference. Twenty-two patients developed VTE in the first six months: nine in the LMWH+ group and 13 in the placebo group [hazard ratio (HR) = 0.51, 95 % confidence interval (CI): 0.19–1.4, P value = 0.29]. At 12 months, there were five (5.1 %) major bleeds in the LMWH+ group and one (1.2 %) on placebo (HR = 4.2, 95 % CI: 0.48–36, P value = 0.22).

A recent retrospective cohort study investigated the effect of systemic LMWH in 30 GBM patients who underwent surgical intervention (resection or biopsy) and subsequent chemo-radiation and adjuvant temozolomide therapy [52]. Thirteen patients received the LMWH enoxaparin (4000 IU/day) for 6 weeks, and 17 did not. The baseline characteristics age, gender, method of surgery and performance status were similar in the two groups. Main endpoints of the study were 1- and 2-year OS, an additional endpoint was progression free survival.

One-year OS was 41.2 % in the LMWH- group and 84.6 % in the LMWH+ patients (P value 0.016). Two-year survival, median OS, and progression free survival were also more favorable in the group that received LWMH, although this difference was not significant. The addition of LMWH did not increase temozolomide toxicity and no DVT or bleeding occurred in either of the groups.

## Discussion

Preclinical studies show an inhibitory effect of heparin and LMWH on GBM growth and angiogenesis. As heparin and LMWH are already widely used in cancer patients, they seem attractive candidates for potential anti-GBM therapy.

Only three studies on the effect of heparin or LMWH in GBM patients have been published [50–52]. The first study showed increased survival in patients who continued dalteparin after first progression [50]. These results could have been influenced by selection, as clinical status determined treatment choice. The PRODIGE trial [51] was terminated early, before significant results could be observed. Zincircioglu et al. showed in a small trial, which included, contrary to the first two studies, patients who received temozolomide in combination with radiotherapy, that 2-year OS was improved by daily injections of LMWH [52]. However, the study groups were not randomly chosen, as the group treated with LMWH had risk factors for VTE, which was the reason and indication for anticoagulant therapy. The non-treatment group lacked such risk factors, making the groups less comparable at baseline. On the other hand it could be stated that the LMWH+ group showed improved OS in spite of their increased risk factors for VTE; a hopeful indication that additional trials should be undertaken.

Studies have attempted to define precise risks (bleeding, thrombocytopenia) and benefits of different heparin variants in overall cancer treatment. A 2013 Cochrane review renders an overview [6]. Nine described trials included patients with a variety of cancer types and stages, mostly solid tumors. The overall effect of parenteral administered heparin/LMWH therapy on the survival of cancer patients was significant at 24 months, but not at 12 months. At 24 months, the mortality risk ratio for the heparin/LMWH treated group was 0.92 (95 % CI 0.88–0.97). Other meta-analyses show similar results, with a non-significant trend towards a beneficial effect of LMWH on cancer patient survival [7, 53–56].

A potential drawback of the use of heparin as a drug targeting GBM angiogenesis, migration, and growth are the anticoagulant properties of heparin/LMWH. For this reason, most cancer patients will receive heparin/LMWH for a limited period of time. Potentially, these short exposure times could influence the anti-cancer effect, resulting in no concrete survival benefits.

To avoid the anticoagulant effects, and thereby risk of major bleeding, heparin mimetic agents have been developed that lack anticoagulant effects [28, 57–60]. These mimics could possibly be administered in higher concentrations and longer treatment regimes. In several tumor models promising results have been shown. In a human gastric carcinoma mouse model, N-desulfated heparin (lacking anticoagulant effects) was shown to decrease metastasis, tumor angiogenesis and levels of bFGF [57]. A comparison of LMWH enoxaparin and non-anticoagulant LMWH (NA-LMWH) treatment of a B16F10 melanoma mouse model indicated that both drugs reduced lung tumor formation, while only enoxaparin prolonged blood clotting time [58]. Another heparin-like compound, M402, inhibited tumor cell migration and sprouting in vitro and demonstrated a survival benefit in a murine mammary carcinoma model [60]. In a mouse model of pancreatic cancer, sulfated non-anticoagulant heparins (S-NACH) as well as the LMWH tinzaparin inhibited tumor growth and angiogenesis [59]. Prolonged bleeding time and hemorrhage were absent in the S-NACH treated group, in contrast to the tinzaparin treated mice. In contrast, another study on a heparan sulfate antibody (αHS), intended to target HSPGs like heparin does, found that αHS stimulated angiogenesis in primary human ECs [28]. This effect was counteracted by heparin. Beneficial anti-tumor effects of non-anticoagulant heparins in GBM models have not yet been published yet.

Although the preliminary results as described in this review may be promising, a well-designed trial is yet to be conducted. Choice of the study drug is debatable: a commonly used LMWH seems self-evident, as this is the common VTE prophylaxis and LMWHs are already widely prescribed. However, with the possible increased risk of (intracranial) hemorrhage, a non-anticoagulant heparin or heparin mimetics should be taken into consideration. Without the increased bleeding risk, greater liberty exists regarding dosage and a significant therapeutic effect could potentially be achieved.

## Electronic supplementary material

(DOCX 44 kb)

## References

[1] Stupp R, Hegi M, Mason W, van den Bent M, Taphoorn M, Janzer R (2009). Effects of radiotherapy with concomitant and adjuvant temozolomide versus radiotherapy alone on survival in glioblastoma in a randomised phase III study: 5-year analysis of the EORTC-NCIC trial. Lancet Oncol.

[2] Green D, Hull R, Brant R, Pineo G (1992). Lower mortality in cancer patients treated with low-molecular-weight versus standard heparin [letter]. Lancet.

[3] Prandoni P, Lensing AW, Büller HR, Carta M, Cogo A, Vigo M (1992). Comparison of subcutaneous low-molecular-weight heparin with intravenous standard heparin in proximal deep-vein thrombosis. Lancet.

[4] Hull RD, Raskob GE, Pineo GF, Green D, Trowbridge AA, Elliott CG (1992). Subcutaneous low-molecular-weight heparin compared with continuous intravenous heparin in the treatment of proximal-vein thrombosis. N Engl J Med.

[5] Lee A, Levine M, Baker R, Bowden C, Kakkar A, Prins M, Rickles F, Julian J, Math M, Haley S, Kovacs M, Gent M (2003). Low-molecular-weight heparin versus a coumarin for the prevention of recurrent venous thromboembolism in patients with cancer. N Engl J Med.

[6] Akl E, Gunukula S, Barba M, Ved Y, Van Doormaal F, Kuipers S, et al (2013) Parenteral anticoagulation in patients with cancer who have no therapeutic or prophylactic indication for anticoagulation (Review). Cochrane database Syst Rev 4:CD006652 10.1002/14651858.CD006652.pub3 21491396

[7] Zacharski LR (2008). Heparin as an anticancer therapeutic. Expert Opin Investig Drugs.

[8] EL Andaloussi S, Mäger I, Breakefield XO, Wood MJ (2013). Extracellular vesicles: biology and emerging therapeutic opportunities. Nat Rev Drug Discov.

[9] Skog J, Würdinger T, van Rijn S, Meijer DH, Gainche L, Sena-Esteves M (2008). Glioblastoma microvesicles transport RNA and proteins that promote tumour growth and provide diagnostic biomarkers. Nat Cell Biol.

[10] Atai NA, Balaj L, van Veen H, Breakefield XO, Jarzyna PA, Van Noorden CJF (2013). Heparin blocks transfer of extracellular vesicles between donor and recipient cells. J Neurooncol.

[11] Zacharski LR, Ornstein DL (1998). Heparin and cancer. Thromb Haemost.

[12] Mousa SA, Petersen LJ (2009). Anti-cancer properties of low-molecular-weight heparin: preclinical evidence. Thromb Haemost.

[13] Hirsh J (1991). Heparin. N Engl J Med.

[14] Barrowcliffe T (2012). History of heparin. Handb Exp Pharmacol.

[15] Bijsterveld N, Hettiarachchi R, Peters R, Prins M, Levi M, Büller H (1999). Low-molecular weight heparins in venous and arterial thrombotic disease. Thromb Haemost.

[16] Petitou M, Casu B, Lindahl U (2003). 1976–1983, a critical period in the history of heparin: the discoveryof the antithrombin binding site. Biochimie.

[17] Gray E, Hogwood J, Mulloy B (2012). The anticoagulant and antithrombotic mechanisms of heparin. Handb Exp Pharmacol.

[18] Magnus N, Gerges N, Jabado N, Rak J (2013). Coagulation-related gene expression profile in glioblastoma is defined by molecular disease subtype. Thromb Haemost.

[19] Magnus N, D’Asti E, Meehan B, Garnier D, Rak J (2014). Oncogenes and the coagulation system-forces that modulate dormant and aggressive states in cancer. Thromb Res.

[20] Sartori MT, Della Puppa A, Ballin A, Campello E, Radu CM, Saggiorato G, d’Avella D, Scienza R, Cella G, Simioni P (2013). Circulating microparticles of glial origin and tissue factor bearing in high-grade glioma: a potential prothrombotic role. Thromb Haemost.

[21] Lever R, Page CP (2012). Non-anticoagulant effects of heparin: an overview. Handb Exp Pharmacol.

[22] Oremus M, Hanson MD, Whitlock R, Young E, Archer C, Dal Cin A (2007). A systematic review of heparin to treat burn injury. J Burn Care Res.

[23] Smorenburg SM, van Noorden CJF (2001). The Complex Effects of Heparins on Cancer. Pharmacol Rev.

[24] Niers TMH, Klerk CPW, DiNisio M, Van Noorden CJF, Büller HR, Reitsma PH (2007). Mechanisms of heparin induced anti-cancer activity in experimental cancer models. Crit Rev Oncol Hematol.

[25] Kargiotis O, Rao JS, Kyritsis AP (2006). Mechanisms of angiogenesis in gliomas. J Neurooncol.

[26] Jain RK, di Tomaso E, Duda DG, Loeffler JS, Sorensen AG, Batchelor TT (2007). Angiogenesis in brain tumours. Nat Rev Neurosci.

[27] Akaogi K, Okabe Y, Sato J, Nagashima Y, Yasumitsu H, Sugahara K (1996). Specific accumulation of tumor-derived adhesion factor in tumor blood vessels and in capillary tube-like structures of cultured vascular endothelial cells. Proc Natl Acad Sci USA.

[28] Christianson HC, van Kuppevelt TH, Belting M (2012). ScFv anti-heparan sulfate antibodies unexpectedly activate endothelial and cancer cells through p38 MAPK: implications for antibody-based targeting of heparan sulfate proteoglycans in cancer. PLoS ONE.

[29] Svensson KJ, Kucharzewska P, Christianson HC, Sköld S, Löfstedt T, Johansson MC (2011). Hypoxia triggers a proangiogenic pathway involving cancer cell microvesicles and PAR-2-mediated heparin-binding EGF signaling in endothelial cells. Proc Natl Acad Sci USA.

[30] Zhao P, Gao C, Dykema K, Furge K, Feng Z, Cao B (2010). Repeated hepatocyte growth factor neutralizing antibody treatment leads to HGF/SF unresponsiveness in human glioblastoma multiforme cells. Cancer Lett.

[31] Lund EL, Olsen MWB, Lipson KE, Mcmahon G, Howlett AR, Kristjansen PEG (2003). Improved Effect of an antiangiogenic tyrosine kinase inhibitor (su5416) by combinations with fractionated radiotherapy or low molecular weight heparin. Neoplasia.

[32] Brat DJ (2003). Malignant glioma physiology: cellular response to hypoxia and its role in tumor progression. Ann Intern Med.

[33] Evans SM, Judy KD, Dunphy I, Jenkins WT, Hwang W-T, Nelson PT (2004). Hypoxia is important in the biology and aggression of human glial brain tumors. Clin Cancer Res.

[34] Belting M, Ahamed J, Ruf W (2005). Signaling of the tissue factor coagulation pathway in angiogenesis and cancer. Arterioscler Thromb Vasc Biol.

[35] Koochekpour S, Jeffers M, Rulong S, Taylor G, Klineberg E, Hudson EA (1997). Met and hepatocyte growth factor/scatter factor expression in human gliomas. Cancer Res.

[36] Vajkoczy P, Menger MD, Vollmar B, Schilling L, Schmiedek P, Hirth KP (1999). Inhibition of tumor growth, angiogenesis, and microcirculation by the novel Flk-1 inhibitor SU5416 as assessed by intravital multi-fluorescence videomicroscopy. Neoplasia.

[37] D’Asti E, Garnier D, Lee TH, Montermini L, Meehan B, Rak J (2012). Oncogenic extracellular vesicles in brain tumor progression. Front Physiol.

[38] De Vrij J, Maas SL, Kwappenberg KM, Schnoor R, Kleijn A, Dekker L, Luider TM, de Witte LD, Litjens M, van Strien ME, Hol EM, Kroonen J, Robe PA, Lamfers ML, Schilham MW (2015). Broekman ML (2015) Glioblastoma-derived extracellular vesicles modify the phenotype of monocytic cells. Int J Cancer.

[39] Al-Nedawi K, Meehan B, Micallef J, Lhotak V, May L, Guha A (2008). Intercellular transfer of the oncogenic receptor EGFRvIII by microvesicles derived from tumour cells. Nat Cell Biol.

[40] Christianson HC, Svensson KJ, Van Kuppevelt TH, Li J, Belting M (2013). Cancer cell exosomes depend on cell-surface heparan sulfate proteoglycans for their internalization and functional activity. PNAS.

[41] Giese A, Westphal M (1996) Glioma invasion in the central nervous system. Neurosurgery 39(2):235–50; discussion 250–2 10.1097/00006123-199608000-00001 8832660

[42] De Aguiar C, Lobão-Soares B, Alvarez-Silva M, Trentin AG (2005). Glycosaminoglycans modulate C6 glioma cell adhesion to extracellular matrix components and alter cell proliferation and cell migration. BMC Cell Biol.

[43] Balzarotti M, Fontana F, Marras C, Boiardi A, Croci D, Ciusani E (2006). In vitro study of low molecular weight heparin effect on cell growth and cell invasion in primary cell cultures of high-grade gliomas. Oncol Res.

[44] Okumura N, Takimoto K, Okada M, Nakagawa H (1989). C6 glioma cells produce basic fibroblast growth factor that can stimulate their own proliferation. J Biochem.

[45] Stukel JM, Caplan MR (2009). Targeted drug delivery for treatment and imaging of glioblastoma multiforme. Expert Opin Drug Deliv.

[46] Rudling MJ, Collins VP, Peterson CO, Pathway LL (1983). Delivery of aclacinomycin a to human glioma cells in vitro by the low-density lipoprotein pathway. Cancer Res.

[47] Goldstein JL, Basu SK, Brunschede GY, Brown MS (1976). Release of low density lipoprotein from its cell surface receptor by sulfated glycosaminoglycans. Cell.

[48] Maguire CA, Gianni D, Meijer DH, Shaket LA, Wakimoto H, Rabkin SD (2010). Directed evolution of adeno-associated virus for glioma cell transduction. J Neurooncol.

[49] Mietzsch M, Broecker F, Reinhardt A, Seeberger PH, Heilbronn R (2014). Differential adeno-associated virus serotype-specific interaction patterns with synthetic heparins and other glycans. J Virol.

[50] Robins HI, O’Neill A, Gilbert M, Olsen M, Sapiente R, Berkey B (2008). Effect of dalteparin and radiation on survival and thromboembolic events in glioblastoma multiforme: a phase II ECOG trial. Cancer Chemother Pharmacol.

[51] Perry JR, Julian JA, Laperriere NJ, Geerts W, Agnelli G, Rogers LR (2010). PRODIGE: a randomized placebo-controlled trial of dalteparin low-molecular-weight heparin thromboprophylaxis in patients with newly diagnosed malignant glioma. J Thromb Haemost.

[52] Zincircioglu SB, Kaplan MA, Isikdogan A, Cil T, Karadayi B, Dirier A (2012). Contribution of low-molecular weight heparin addition to concomitant chemo- radiotherapy in the treatment of glioblastoma multiforme. J Balk Union Oncol.

[53] Che DH, Cao JY, Shang LH, Man YC, Yu Y (2013). The efficacy and safety of low-molecular-weight heparin use for cancer treatment: a meta-analysis. Eur J Intern Med.

[54] Kuderer NM, Ortel TL, Francis CW (2009). Impact of venous thromboembolism and anticoagulation on cancer and cancer survival. J Clin Oncol.

[55] Sanford D, Naidu A, Alizadeh N, Lazo-Langner A (2014). The effect of low molecular weight heparin on survival in cancer patients: an updated systematic review and meta-analysis of randomized trials. J Thromb Haemost.

[56] Lazo-Langner A, Goss G, Spaans J, Rodger M (2007). The effect of low- molecular-weight heparin on cancer survival. A systematic review and meta- analysis of randomized trials. J Thromb Haemost.

[57] Chen J-L, Fan J, Chen M-X, Dong Y, Gu J-Z (2012). Effect of non-anticoagulant N-desulfated heparin on basic fibroblast growth factor expression, angiogenesis, and metastasis of gastric carcinoma in vitro and in vivo. Gastroenterol Res Pract.

[58] Mousa SA, Linhardt R, Francis JL, Amirkhosravi A (2006). Anti-metastatic effect of a non-anticoagulant low-molecular-weight heparin versus the standard low-molecular-weight heparin, enoxaparin. Thromb Haemost.

[59] Sudha T, Yalcin M, Lin H-Y, Elmetwally AM, Nazeer T, Arumugam T (2014). Suppression of pancreatic cancer by sulfated non-anticoagulant low molecular weight heparin. Cancer Lett.

[60] Zhou H, Roy S, Cochran E, Zouaoui R, Chu CL, Duffner J (2011). M402, a novel heparan sulfate mimetic, targets multiple pathways implicated in tumor progression and metastasis. PLoS ONE.

