# Supplementary material for: Heparin in malignant glioma: review of preclinical studies and clinical results
Source: J Neurooncol. 2015 Jun 30;124(2):151–6. doi: 10.1007/s11060-015-1826-x (PMC4582077; doi:10.1007/s11060-015-1826-x)
Supplement: Supplementary file 1 — (DOCX 44 kb) [file 11060_2015_1826_MOESM1_ESM.docx]

Supplementary table 1 Search terms

|  | Search terms used Search Date: 1 April 2015 | Number of articles |
| --- | --- | --- |
| Pubmed | heparin[tiab] OR heparins[tiab] OR heparinic[tiab] OR heparinate[tiab] OR UFH[tiab] OR Dalteparin[tiab] OR Bemiparin[tiab] OR Certoparin[tiab] OR Nadroparin[tiab] OR Parnaparin[tiab] OR Reviparin[tiab] OR Tinzaparin[tiab] OR Enoxaparin[tiab] OR LMWH[tiab] OR LMWHs[tiab] | **360** |
|  | AND |  |
|  | glioblastoma[tiab] OR glioblastomas[tiab] OR glioma[tiab] OR gliomas[tiab] OR GBM[tiab] OR ((brain[tiab] OR “central nervous”[tiab] OR cranial[tiab] OR intracranial[tiab]) AND (cancer[tiab] OR cancers[tiab] OR tumor[tiab] OR tumors[tiab] OR tumour[tiab] OR tumours[tiab])) |  |
| Embase | (heparin:ti:ab OR heparins:ti:ab OR heparinic:ti:ab OR heparinate:ti:ab OR UFH:ti:ab OR Dalteparin:ti:ab OR Bemiparin:ti:ab OR Certoparin:ti:ab OR Nadroparin:ti:ab OR Parnaparin:ti:ab OR Reviparin:ti:ab OR Tinzaparin:ti:ab OR Enoxaparin:ti:ab OR LMWH:ti:ab OR LMWHs:ti:ab) | **483** |
|  | AND |  |
|  | (glioblastoma:ti:ab OR glioblastomas:ti:ab OR glioma:ti:ab OR gliomas:ti:ab OR GBM:ti:ab OR ((brain:ti:ab OR “central nervous”:ti:ab OR cranial:ti:ab OR intracranial:ti:ab) AND (cancer:ti:ab OR cancers:ti:ab OR tumor:ti:ab OR tumors:ti:ab OR tumour:ti:ab OR tumours:ti:ab))) |  |

Supplementary table 1: Search terms as used in Pumbed and Embase databases. Updated search date: April 1^st^, 2015.

Supplementary figure 1 Flowchart

PubMed
**360**

Embase
**483**

*All decisions were by consensus of two authors

Search date: 01 April 2015

Screening title/ abstract *
**105**

Filtering doubles
**521**

**Exclusion criteria:**

- Non English/Dutch/ Germna/French
- Case reports
- Expert opinion
- Meeting abstract

Full text available
**86**

Useful articles
**15**

Related articles
**3**

**Inclusion criteria:**

- Heparin treatment studies
- Original studies
- Human/animal/ preclinical

**Domain** AND **Determinant**

**Exclusion criteria:**

- No heparin treatment
- Not enough information in paper

Cross reference
**4**

Supplementary figure 1: Flowchart illustrating the process of systematic search, indicating reasons to eliminate articles from this review.
